# Dynamic Tracking Human Mesenchymal Stem Cells Tropism following Smoke Inhalation Injury in NOD/SCID Mice

**DOI:** 10.1155/2016/1691856

**Published:** 2016-09-20

**Authors:** MeiJuan Song, Qi Lv, XiuWei Zhang, Juan Cao, ShuLi Sun, PeiXin Xiao, ShiKe Hou, Hui Ding, ZiQuan Liu, WenLong Dong, JinQiang Wang, Xue Wang, ZhiGuang Sun, Man Tian, HaoJun Fan

**Affiliations:** ^1^Respiratory Department, Affiliated Jiangning Hospital, Nanjing Medical University, Jiangsu, China; ^2^Institute of Disaster Medicine and Public Health, Affiliated Hospital of Logistic University of Chinese People's Armed Police Force, Tianjin 300162, China; ^3^Respiratory Department, Affiliated Nanjing Children's Hospital, Nanjing Medical University, Jiangsu, China

## Abstract

Multiple preclinical evidences have supported the potential value of mesenchymal stem cells (MSCs) for treatment of acute lung injury (ALI). However, few studies focus on the dynamic tropism of MSCs in animals with acute lung injury. In this study, we track systemically transplanted human bone marrow-derived mesenchymal stem cells (hBMSCs) in NOD/SCID mice with smoke inhalation injury (SII) through bioluminescence imaging (BLI). The results showed that hBMSCs systemically delivered into healthy NOD/SCID mouse initially reside in the lungs and then partially translocate to the abdomen after 24 h. Compared with the uninjured control group treated with hBMSCs, higher numbers of hBMSCs were found in the lungs of the SII NOD/SCID mice. In both the uninjured and SII mice, the BLI signals in the lungs steadily decreased over time and disappeared by 5 days after treatment. hBMSCs significantly attenuated lung injury, elevated the levels of KGF, decreased the levels of TNF-*α* in BALF, and inhibited inflammatory cell infiltration in the mice with SII. In conclusion, our findings demonstrated that more systemically infused hBMSCs localized to the lungs in mice with SII. hBMSC xenografts repaired smoke inhalation-induced lung injury in mice. This repair was maybe due to the effect of anti-inflammatory and secreting KGF of hMSCs but not associated with the differentiation of the hBMSCs into alveolar epithelial cells.

## 1. Introduction

Smoke inhalation injury (SII) is caused by smoke-induced damage of the respiratory tract and lung parenchyma, with or without additional heat-induced damage. SII is a major cause of morbidity and mortality in victims of fire tragedies [1], affecting approximately 22% of all burn patients and resulting in at least 30% of all fire-related mortality [2]. Moreover, 80% to 90% of fire-related fatalities have been attributed to smoke inhalation [3]. The major harmful components of smoke include heat, systemic toxins (e.g., CO and cyanide), and respiratory irritants [4], which damage the respiratory tract and lung tissue. This results in laryngeal/pulmonary edema, airway obstruction, and ventilation/perfusion mismatch [5]. Severe cases may develop acute respiratory distress syndrome (ARDS) [3]. Determining the best method for treating burn victims, especially during the early stages of smoke inhalation-induced acute lung injury (ALI), remains to be a difficult problem in the field of first aid medicine. Current treatments for SII mainly focus on oxygen administration, airway management, fluid resuscitation, mechanical ventilation, and the use of specific medications [1, 4, 6]. Although many drugs are effective in reducing lung injury in animal models, only a few drugs, including anticoagulants, *β*2-agonists, antioxidants, and inflammatory mediator agonists, are currently applied in the clinical setting [6, 7].

Mesenchymal stem cells (MSCs) are self-renewing, multipotent progenitor cells that have the potential to differentiate into multiple different mesodermal lineages. It has been shown in many different animal models that MSCs have a remarkable ability to localize to sites of injury and exert nonimmunogenic and immunosuppressive characteristics [8, 9]. Based on these properties, MSCs offer a promising source for cell-based treatment of various complicated disorders, such as graft-versus-host disease [10, 11], cardio/cerebrovascular disease [12], spinal cord injury [13], hepatic disease [14], and respiratory disease [15, 16]. Furthermore, many studies have indicated that MSCs exert protective effects against ALI via their secretion of multiple paracrine factors, including endothelial and epithelial growth factors, anti-inflammatory cytokines, and antimicrobial peptides [17–20]. However, these studies have mainly focused on endotoxin-induced ALI, and researches focusing on the effects of MSCs on smoke inhalation-induced ALI still lack. Furthermore, uncertainties remain regarding the localization and persistence of MSCs in vivo following their administration into subjects with ALI.

Bioluminescent imaging (BLI), a recently developed technique that enables the noninvasive study of ongoing biological processes in small laboratory animals, can be used to track luciferase- (Luc-) expressing cells implanted into living animals in real time. In a previous study, Kidd et al. used BLI to track the dynamic distribution of firefly Luc-expressing human MSCs (hMSCs) following their systemic injection into healthy mice, mice subjected to inflammatory insults, and mice bearing tumors. The hMSCs were found to initially localize to the lungs and later moved into the liver and spleen. Additionally, the Luc signal produced by the hMSCs decreased over time. In wounded mice and tumor-bearing mice, the hMSCs were found to localize to injured tissue or tumors after systemic administration [21]. Although it has been shown that MSCs initially localize to the lungs following systemic delivery, studies examining the dynamic distribution of MSCs after their intravenous injection into mice with SII are lacking. In the current study, we modified human bone marrow-derived MSCs (hBMSCs) to stably coexpress Luc and green fluorescent protein (GFP) reporter genes (Luc-GFP-hBMSCs). We then used BLI to track the dynamics of the cells' localization patterns for 14 days following their systemic administration into normal mice and mice with SII. Our results provide experimental support for the use MSCs to treat SII.

## 2. Materials and Methods

### 2.1. Cells and Animals

hMSCs were purchased from Cyagen Biosciences (Guangzhou, China) and grown in Dulbecco's modified Eagle's medium (DMEM) (Cyagen Biosciences) supplemented with 10% heat-inactivated fetal bovine serum (FBS) (Cyagen) at 37°C under 5% CO_2_.

Male NOD/SCID mice, aged between 6 and 8 weeks and ranging in weight from 25 to 30 g, were purchased from Wei Tong Li Hua Experimental Animal Technology Co., Ltd (Beijing, China). The mice were used in accordance with institutional guidelines and following approved protocols.

### 2.2. Lentiviral Vector Construction and MSC Transduction

Lentiviral vectors carrying a Luc and GFP dual-fusion reporter gene were constructed and purified by Shanghai GeneChem Co., Ltd. For transduction, hMSCs were seeded into 25 cm^2^ flasks containing appropriate growth medium and grown to 20%–30% confluence. Then, the GFP-Luc lentivirus vectors were added at a multiplicity of infection (MOI) of 10 to a 2.5 mL aliquot of hMSCs in growth medium containing 5 *μ*g/mL polybrene. The cells were incubated with the viruses for 8–12 h, after which fresh medium was added to each flask, and the cells were incubated for an additional 48–72 h. The cells were passaged 1 : 2 and grown to 80–100% confluence. Three days after transduction, the cells were viewed on a Leica DMI4000 inverted microscope equipped with a fluorescence source and a charge-coupled device (CCD) camera. Transduction efficiency was determined by fluorescence-activated cell sorting (FACS) analysis of GFP expression using previously described settings [22].

### 2.3. Flow Cytometry Analysis

Luc-GFP-hBMSCs were harvested with 0.25% trypsin-EDTA and resuspended in phosphate-buffered saline (PBS) supplemented with 2% FBS. Approximately 1 × 10^6^ cells were stained with 1 *μ*g of antibody for 30 minutes at 4°C and then analyzed on a FACS Caliber flow cytometer (Becton Dickinson, Franklin Lakes, NJ). Human antibodies against the following proteins were used for this analysis: CD105, CD29, CD73, CD44, CD90, CD34, CD45, and CD11c (BD Biosciences).

### 2.4. Multilineage Differentiation of Transduced hMSCs

To determine the multilineage differentiation potential of the transduced hMSCs, we cultured the cells in various types of differentiation media according to manufacturer recommendations (Cyagen Biosciences, Guangzhou, China, http://www.cyagen.com/). To induce adipogenic differentiation, Luc-GFP-hBMSCs were subcultured in six-well plates at 2 × 10^4^ cells/cm^2^ in growth medium containing 10% FBS, 5% penicillin-streptomycin, and 2 mM L-glutamine. The culture medium was replaced every 3 days until the cells reached 100% confluence, after which the growth medium was replaced with induction medium (2 mL per well) containing FBS, penicillin-streptomycin, glutamine, insulin, rosiglitazone, and dexamethasone. Three days later, the medium was replaced with maintenance medium consisting of FBS, penicillin-streptomycin, and insulin. After 24 h, the maintenance medium was changed back to induction medium, and this cycle was repeated three times. After five cycles of induction/maintenance, the cells were cultured in maintenance medium for 3 days. Three weeks later, adipose cells were stained for visualization with Oil Red O.

To induce osteogenic differentiation, Luc-GFP-hBMSCs were cultured in growth medium at a density of 3 × 10^4^ cells/cm^2^ for 1 day at 37°C in a 5% CO_2_ humidified incubator. Following this, the growth medium was aspirated and replaced with osteogenic differentiation medium (2 mL per well) containing FBS, penicillin-streptomycin, glutamine, ascorbate, *β*-glycerophosphate, and dexamethasone. The medium was replaced every three days. Three weeks later, the cells were fixed with 2 mL of 4% formaldehyde solution and stained with Alizarin red. A light microscope was used to visualize and capture images of the stained cells.

To induce chondrogenic differentiation, human MSCs at subconfluent conditions were trypsinized and aliquots of 2 × 10^5^ cells per well were added to a 15 mL centrifuge tube, and the plate was spun at 400 ×g for 5 min. For differentiation into chondrocytes, cells were cultured in a commercialized chondrogenic induction medium in the presence of 10 ng/mL recombinant human TGF-*β*3. The cell pellets formed free-floating aggregates within the first 24 h. The medium was replaced every 2-3 days, and aggregates were cultured for 28 days and collected for paraffin section following Alcian Blue staining.

### 2.5. Establishment of Smoke Inhalation Mouse Model

All animals used in this study received humane care in compliance with the Guide for the Care and Use of Laboratory Animals published by the National Institutes of Health. The study protocol was approved by the Laboratory Animal Ethics Committee of the Affiliated Hospital of Logistical College of Chinese People's Armed Police Forces. All surgeries were performed under sodium pentobarbital anesthesia, and all efforts were made to minimize suffering.

Models of SII were established using a previously described device that was constructed in-house [23]. To accomplish this, awake male NOD/SCID mice were exposed to combustion smoke generated by smoldering wood shavings in a smoke-generating container connected to a 20 L transparent exposure chamber. The mice were subjected to the smoke for 0, 3, 5, 7, and 9 min. The establishment of severe SII was assessed by blood carboxyhemoglobin (COHb) concentration, blood gas analysis, measurement of the wet/dry (W/D) weight ratio of lung tissue, and lung histopathology. Blood was collected from a subset of mice that were killed 1 h after smoke exposure to measure COHb concentration with an oximeter (482 CO-Oximeter) [24] and analyze blood gas content using a Radiometer ABL 625 Blood Gas Analyzer (Copenhagen, Denmark) [25]. Another subset of mice were killed 3 d after smoke exposure, and their lungs were isolated to measure W/D ratios and for histological analysis.

### 2.6. Luc-GFP-hBMSC Administration and Bioluminescent Imaging

At 24 h after smoke inhalation, 100 *μ*L aliquots of Luc-GFP-hBMSCs (3 × 10^5^ cells) were injected into the tail veins of control and SII NOD/SCID mice. The mice were then submitted to BLI to visualize the localization of the Luc-GFP-hBMSCs at 1.5, 2.5, 5, 7.5, 10, and 24 h and 3 and 5 days after injection [21].

To monitor Luc-GFP-hBMSC localization to the lungs, we submitted mice to isoflurane anesthesia and then intraperitoneally injected them with D-luciferin firefly potassium salt substrate (150 mg/kg body weight in 100 *μ*L PBS). Then, we placed the animals into an IVIS system in a supine position (Caliper Life Sciences, Hopkinton, MA). The animals were imaged over a 10 min time period with 1 min acquisition intervals [26]. To quantify light emission, a region of interest (ROI) was manually selected based on signal intensity. The area of the ROI was kept constant while the signal intensity was recorded as average photons per second per square centimeter per steradian as previously described [21].

### 2.7. Analysis of Wet/Dry Weight Ratio of Lung Tissue

After treatment as described above, the mice were killed, and their left lungs were isolated. After blotting off blood and other contaminants, the wet weights of the lung tissue samples were measured. Then, the lungs were dried in a 70°C oven for 72 h, and their dry weights were measured. The W/D weight ratios of the lungs were then calculated as previously described [25].

### 2.8. Histology and Immunohistochemistry

The right lungs of the mice treated as described above were isolated, and their upper and middle lobes were fixed in 10% formalin for 24 h. The tissue samples were then dehydrated, embedded in paraffin, and cut into 5 mm thick sections. Following this, the samples were stained with hematoxylin and eosin (H&E) after deparaffinization and evaluated under an optical microscope (Olympus BX51, Japan).

Luc-GFP-hBMSCs in lung tissue were detected by immunostaining for GFP. After deparaffinization and rehydration, paraffin sections were placed into a pressure cooker containing antigen retrieval buffer (0.01 M citrate buffer, pH 6.0) under full pressure for 2 minutes to unmask antigens. Immunostaining was performed by incubating the sections with a rabbit anti-GFP monoclonal antibody (1 : 100, Abcam, MA) overnight at 4°C, followed by incubation with a biotin-conjugated secondary antibody (ZSGB-bio, China) at 37°C for 1 h and horseradish peroxidase-conjugated streptavidin (ZSGB-bio, China) at 37°C for 30 min. The sections were stained with a DAB kit, which were counterstained with hematoxylin to visualize cell nuclei. Images were obtained with an Olympus BX51 microscope, and the proportion of positively stained cells was determined using Image-Pro Plus version 5.1. For histological and immunohistochemical analysis, the slides were labeled with numbers, and double-blinded examinations were performed by two independent pathologists.

### 2.9. Semiquantitative PCR

Total RNA for PCR was extracted using an RNeasy kit (Solarbio, Beijing, China), which included a DNase digestion step to remove any contaminating DNA. Semiquantitative reverse transcription PCR was performed using a thermal cycler (Thermo), and amplified products were visualized using agarose gels. The following primers were used for PCR:

 Luciferase forward: ACTGGGACGAAGACGAACAC.
 Luciferase reverse: GGCGACGTAATCCACGATCT.
 
*β*-actin forward: GTGGGGCGCCCCAGGCACCA.
 
*β*-actin reverse: CTTCCTTAATGTCACGCACGATTTC.

### 2.10. Analysis of TNF-*α* and KGF Levels in Bronchoalveolar Lavage Fluid

Bronchoalveolar lavage (BAL) was performed by instilling and withdrawing sterile physiological saline (1 mL) through a tracheal cannula using a 20-gauge Surflo i.v. catheter. This procedure was repeated three times, and the three BAL fluid (BALF) samples were pooled. The BALF was centrifuged (300 ×g, 5 min), and the supernatant portions were stored at −80°C for further examination. For the detections of TNF-*α* and keratinocyte growth factor (KGF), the supernatant of BALF was analyzed by using mouse TNF-*α* ELISA kit (eBioscience, San Diego, CA, USA) and KGF ELISA kit (Nanjing Jiancheng Bioengineering Institute, Nanjing, China) following the instructions of manufacturer.

### 2.11. Statistical Analysis

All data were processed using SPSS version 13.0 statistical software. The data are shown as the mean ± standard deviation $\left(\overset{-}{x}\pm s\right)$. Sample measurement data between groups were compared using independent samples *t*-tests, and group data were compared using paired *t*-tests. *P* < 0.05 was considered significant.

## 3. Results

### 3.1. hMSC Transduction

**Figure 1:**
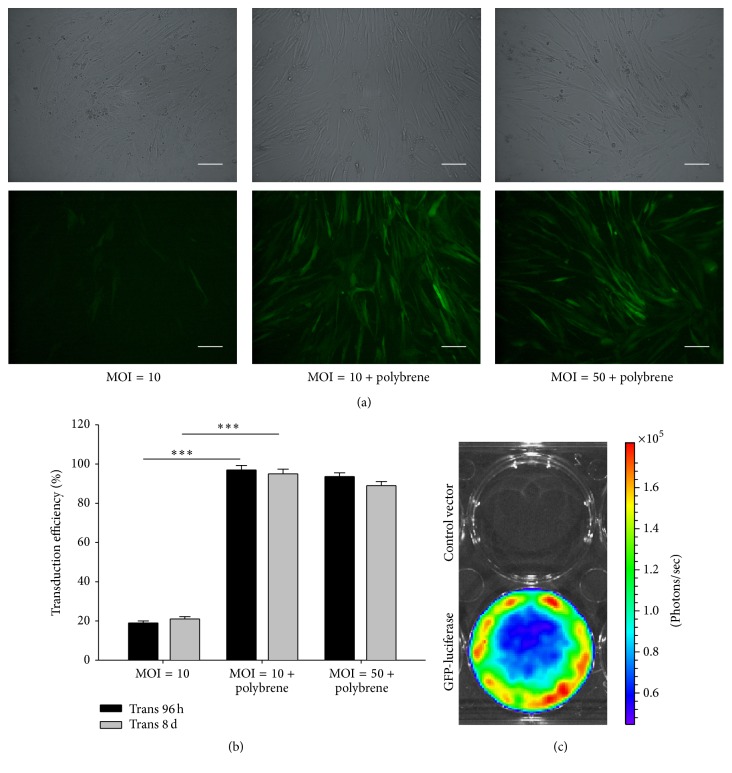
Characterization of human mesenchymal stem cells expressing firefly luciferase and green fluorescent protein reporter genes. (a) Human mesenchymal stem cells (hMSCs) were transduced with a lentiviral vector carrying luciferase (Luc) and green fluorescent protein (GFP) reporter genes, and GFP expression within the cytosol was measured. (b) Transduction efficiencies of Luc-GFP lentiviral vector-transduced hMSCs after 96 h and 8 d. *∗∗∗*  indicates  *P* < 0.01 between the indicated groups. (c) Firefly Luc-expressing cells showed specific activity after treatment with D-luciferin and coelenterazine in vitro.

Lenti-GFP-Luc with a 2.00 E + 8 TU/mL titer was used at an MOI of 10 to infect hMSCs. Transduction efficiency was approximately 90% after 48 h based on fluorescence and phase-contrast microscopy (Figures 1(a) and 1(b)). GFP expression remained stable for at least 30 d under constant culture conditions (data not shown). In vitro Luc activity was assessed using BLI following the application of D-luciferin. Only the transduced cells showed Luc activity and not the control cells (Figure 1(c)).

### 3.2. Characterization of Luc-GFP-hBMSCs

**Figure 2:**
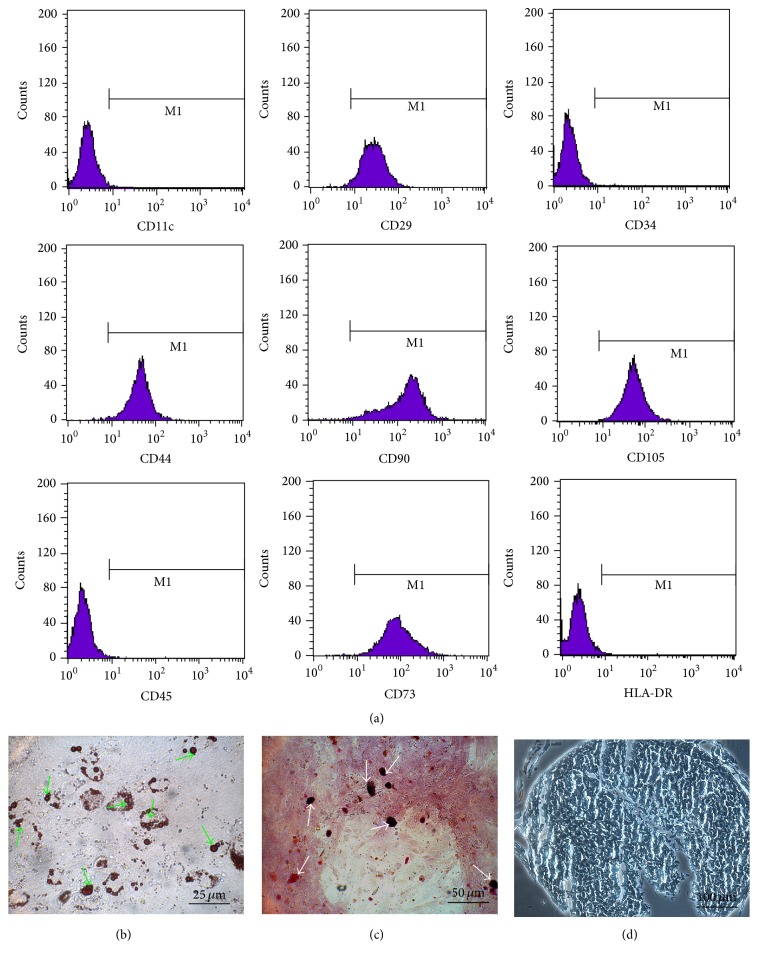
Characterization of human bone marrow-derived stem cells coexpressing luciferase and green fluorescent protein reporter genes (Luc-GFP-hBMSCs). (a) Flow cytometric analysis of Luc-GFP-hBMSCs. Luc-GFP-hBMSCs were harvested with 0.25% trypsin-EDTA and resuspended in phosphate-buffered saline supplemented with 2% fetal bovine serum. Following this, the expression levels of CD90, CD105, CD29, CD73, CD44, CD34, CD45, CD11c, and HLA-DR were measured by fluorescence-activated cell sorting analysis. (b)–(d) Luc-GFP-BMSCs were able to differentiate into adipocytes, osteoblasts, and chondrocytes in vitro as shown by positive Oil Red O staining (b), Alizarin red staining (c), and Alcian Blue staining (d). The green arrows indicate lipid droplets stained with Oil Red O, and the white arrows indicate calcium nodules stained with Alizarin red. Acid mucopolysaccharide of chondrocytes could be stained by Alcian Blue.

After transduction with Lenti-GFP-Luc, FACS analysis demonstrated that Luc-GFP-hBMSCs expressed high levels of CD105, CD29, CD73, CD44, and CD90 and low levels of CD34, CD45, and CD11c. These proteins were chosen for analysis because they represent well-established phenotypic markers for hMSCs (Figure 2(a)) [23]. The expression patterns were consistent across all hMSCs tested, and the tested hMSCs were used in the following experiments. Additionally, we subjected transduced hMSCs to adipogenic and osteoblastic differentiation assays. In all cases, cells positively stained for Oil Red O and Alizarin red were detected after culture in differentiation medium, suggesting that the cells maintained differentiation potential regardless of lentiviral transduction (Figures 2(b) and 2(c)). Furthermore, chondrogenic differentiation of transduced hMSCs was confirmed by staining the acid mucopolysaccharide of chondrocytes with Alcian Blue (Figure 2(d)).

### 3.3. Establishment of Mouse Model of Smoke Inhalation Injury

To establish a mouse model of SII, a smoke generator constructed in-house was utilized, as previously described [23]. To optimize the experimental conditions for inducing SII, mice were subjected to smoke for 0, 3, 5, 7, and 9 min. 85% of mice exposed to smoke for 9 min died of hypoxia, so this time point was excluded in the following detections. COHb concentration and blood gas content were measured immediately after smoke exposure and again 1 h later. As the smoke exposure time increased, the COHb concentration increased, while the PaO_2_ and PaO_2_/FiO_2_ content in blood decreased (Figure 1(a)). The PaO_2_/FiO_2_ ratio was below 300 at 7 min in the SII group, which is within the standard for mild ARDS according to the Berlin Definition [27].

**Figure 3:**
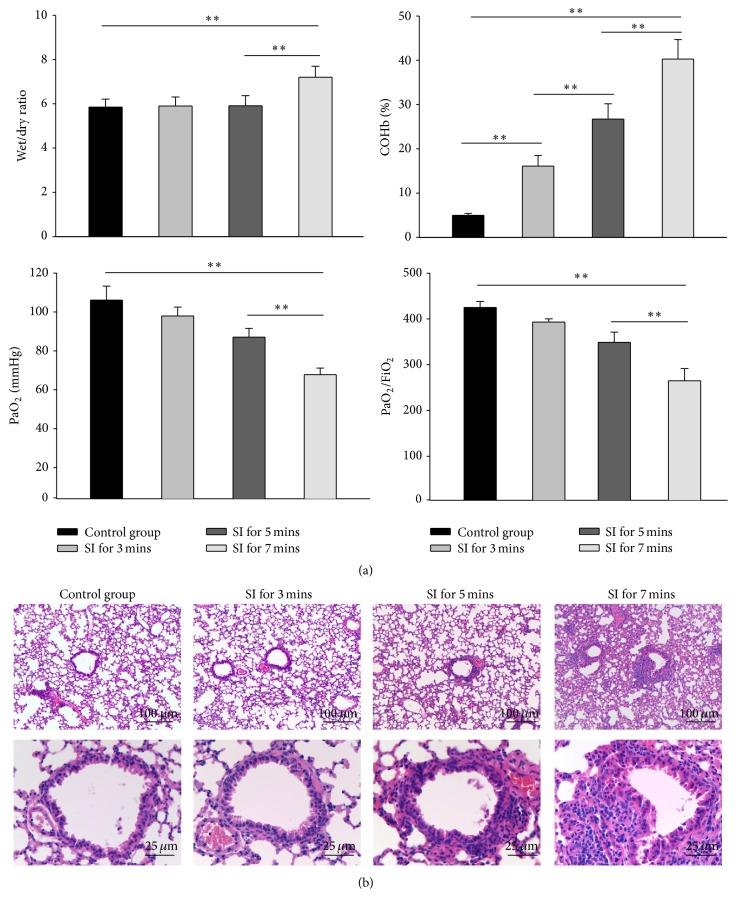
Establishment of smoke inhalation NOD/SCID mouse model. NOD/SCID mice (*n* = 6) were subjected to 0, 3, 5, 7, and 9 min of smoke exposure. (a) Wet/dry (W/D) weight ratios, blood carboxyhemoglobin (COHb), PaO_2_, and PaO_2_/FiO_2_ were measured at the indicated time points. *∗∗*  indicates  *P* < 0.01 between the indicated groups. (b) Representative histological images of lung sections from mice with or without smoke inhalation injury (*n* = 6).

The W/D weight ratio was used as an index of water accumulation in the lung, which is an indicator of lung edema. At 3 d after smoke exposure, the lung W/D ratios in the 7 min SII group were significantly elevated relative to the control group; however, there were no differences noted in the 3 or 5 min SII groups (Figure 3(a)). Accordingly, histopathology results showed that exposure to smoke for 7 min led to the most serious pathological changes in the lungs, including narrowed alveolar space, thickened alveolar and bronchiole walls, and inflammatory cell infiltration around the airway (Figure 3(b)). Based on these results, we used a 7 min smoke exposure protocol for the following studies.

### 3.4. Dynamics of hMSC Distribution after Injection into Mice with SII

**Figure 4:**
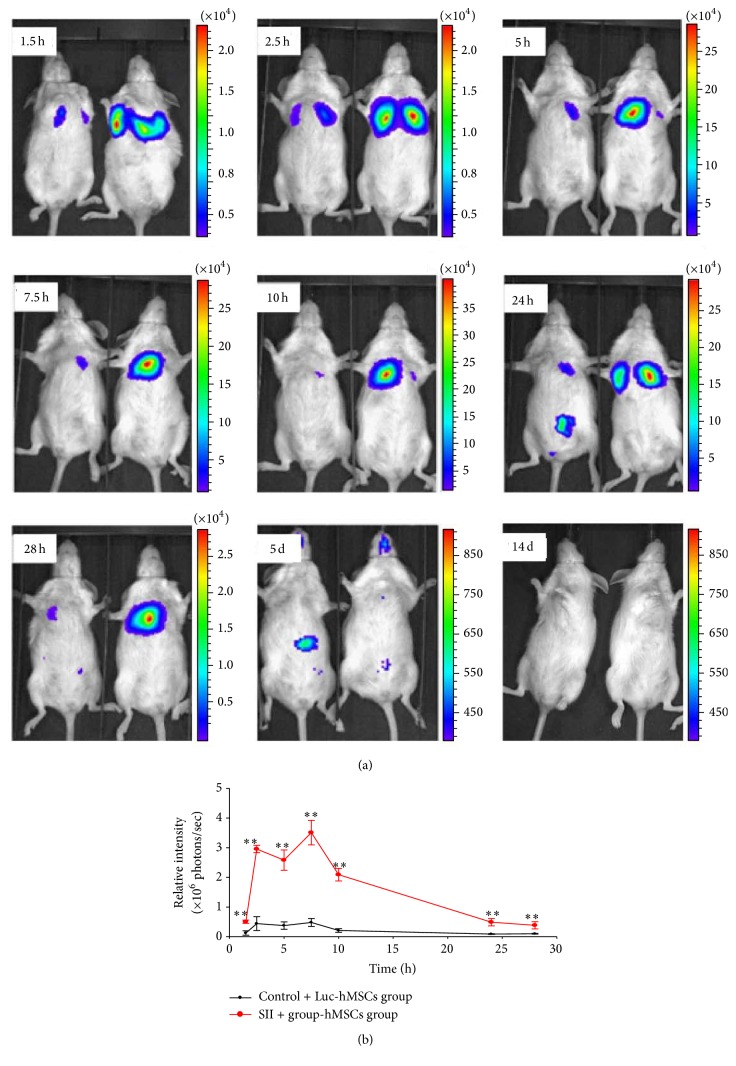
Biodistribution of human bone marrow-derived stem cells coexpressing luciferase and green fluorescent protein reporter genes in mice with or without smoke inhalation injury. Human bone marrow-derived stem cells coexpressing luciferase and green fluorescent protein reporter genes (Luc-GFP-hBMSCs) were intravenously injected into NOD/SCID mice and imaged at 1.5, 2.5, 5, 7.5, 10, 24, and 28 h and 5 and 14 d after injection. (a) The Luc-GFP-hBMSCs initially localized to the lung and then migrated to the abdomen by 24 h. Reporter gene expression completely disappeared by 14 d after injection. (b) Quantification of the bioluminescent signal over this time period indicated that a greater number of systemically infused Luc-GFP-hBMSCs localized to the lungs in mice with smoke inhalation injury compared to uninjured mice (*n* = 6). *∗∗*  indicates  *P* < 0.01 compared with the uninjured control group treated with Luc-GFP-hBMSCs.

Luc-GFP-hBMSCs (3 × 10^5^ cells/animal) were intravenously injected into NOD/SCID mice with or without SII. Live-animal BLI was then used to monitor Luc-GFP-hBMSC localization patterns over time. As shown in Figure 4, luciferase expression was initially detected in the lungs in both the control and the SII plus Luc-hBMSC groups (Figure 4(a)). Compared with the control group, stronger BLI signals were produced in the SII group. The BLI signals in the lungs peaked at 7.5 h after infusion of hBMSCs and then gradually diminished with time (Figure 4(b)). At 24 h after infusion of hBMSCs, the bioluminescent signals began to shift to the abdomen in the control group. However, the signals were still primarily in the lungs in the mice with SII. Five days after injection of hBMSCs, the BLI signals disappeared completely from the lungs, and the abdomens of the mice in the control group showed only weak BLI signals. Fourteen days after injection, no signals were detected in either the control group or the SII group (Figures 4(a) and 4(b)).

### 3.5. PCR and Immunohistochemistry Detection of hBMSC Engraftment

**Figure 5:**
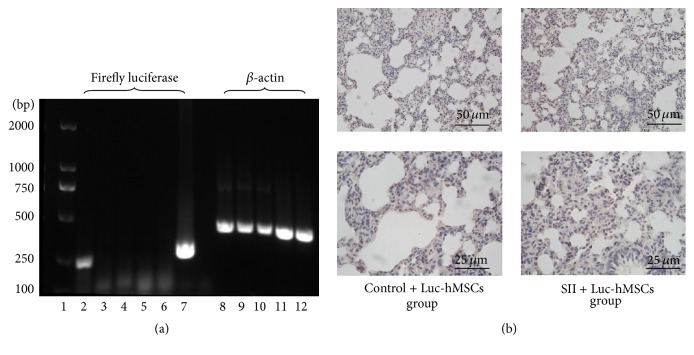
Detection of human bone marrow-derived stem cells coexpressing luciferase and green fluorescent protein reporter genes in mouse tissues at 14 days after injection. (a) Expression of the firefly luciferase gene was detected by PCR. *β*-actin was used as a loading control. Panel 1: DNA marker. Panels 2–7: from left to right, human bone marrow-derived stem cells coexpressing luciferase and green fluorescent protein reporter genes (Luc-GFP-hBMSCs), liver from a control mouse treated with Luc-GFP-hBMSCs, liver from a mouse with smoke inhalation injury (SII) treated with Luc-GFP-hBMSCs, lung from a control mouse treated with Luc-GFP-hBMSCs, and lung from a mouse with SII treated with Luc-GFP-hBMSCs. Panels 8–12: corresponding *β*-actin levels from panels 2–6. Representative images from at least three independent experiments. (b) Luc-GFP-hBMSCs were detected by immunohistochemistry (anti-GFP) in the lung at day 14 after injection into mice with SII (*n* = 6).

Fourteen days after the injection of Luc-GFP-hBMSCs, the injected mice were sacrificed, and PCR was used to detect Luc expression in liver and lung tissues. Consistent with the BLI results described above, no Luc expression was detected in either the control group or the SII group that underwent hBMSC administration (Figure 5(a)). Accordingly, no GFP-positive cells were found in the lung tissue samples collected from these mice (Figure 5(b)).

### 3.6. Protective Effects of hBMSC Xenografts in SII Mice

**Figure 6:**
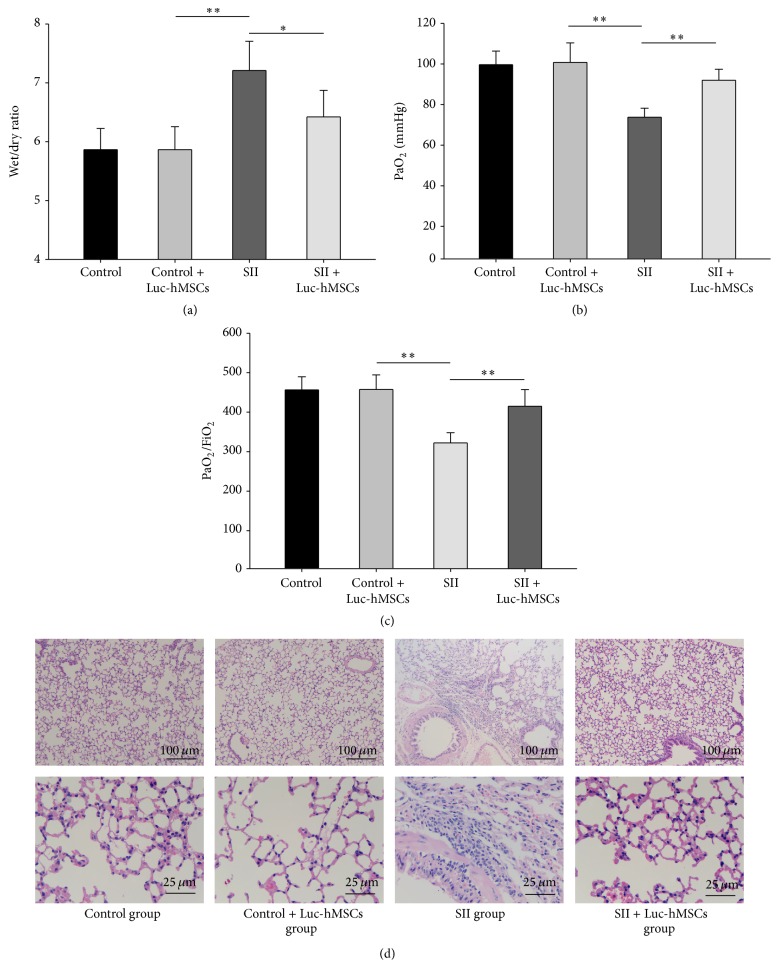
Evaluation of the protective effects of hMSCs against smoke inhalation lung injury in mice. (a)–(c) Wet/dry (W/D) weight ratios, PaO_2_, and PaO_2_/FiO_2_ were measured at 14 days after injection of hBMSCs into mice with or without SII (*n* = 6). ^*∗*^ *P* < 0.05, ^*∗∗*^ *P* < 0.01 between the indicated groups. (d) Representative pictures of histological examinations of lung sections from the indicated groups.

Fourteen days after the injection of hBMSCs into mice with SII, we evaluated the effects produced by the cells by measuring lung W/D weight ratios and evaluating pathological changes in lung tissue samples. There were no significant differences in W/D weight ratio in the control group before and after injection. The lung W/D ratio in the SII + hBMSC group was significantly lower than that in the untreated SII group (*P* < 0.01), indicating that pretreatment with hBMSCs could decrease the degree of lung edema produced by smoke inhalation (Figure 6(a)). Moreover, PaO_2_ and PaO_2_/FiO_2_ were significantly improved after hBMSCs treatment compared with SII group (*P* < 0.01) (Figures 6(b) and 6(c)).

As shown in Figure 6(d), there were no obvious differences in lung tissue samples collected from mice treated with hBMSCs compared to untreated mice. In the SII group, the alveolar walls burst, and the alveolar space was narrowed. In addition, there was significant infiltration of polymorphonuclear leukocytes (PMNs) around the airway. The administration of hBMSCs markedly reduced the severity of pulmonary injury induced by smoke inhalation. In the lungs of the mice in the SII + hBMSCs group, the alveolar space was widened, there was less PMN infiltration, and there were thinner alveolar septa compared to the lungs of the untreated mice with SII. These results indicated that the hBMSC xenografts protected mice from damage associated with SII.

### 3.7. Analysis of KGF and TNF-*α* Levels in BALF

**Figure 7:**
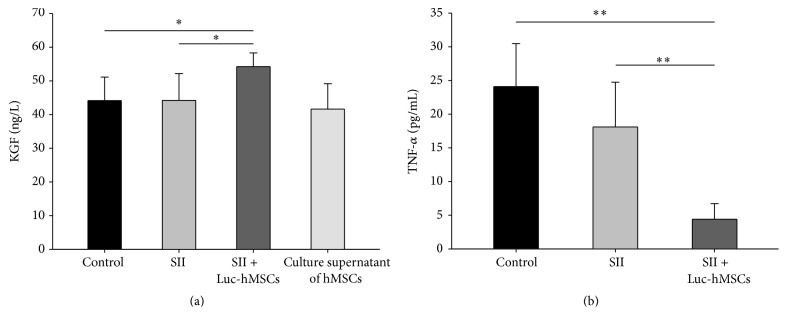
The effects of hMSCs treatment on the levels of TNF-*α* and KGF in BALF of mice with SII. At one day after injection of hMSCs, (a) the concentrations of keratinocyte growth factor (KGF) in the BALF were measured for control, SII, and Luc-hMSCs treatment groups. The culture supernatant of hBMSC was used as a positive control. (b) The concentrations of TNF-*α* in the BALF were measured for the indicated groups (*n* = 6). ^*∗*^ *P* < 0.05, ^*∗∗*^ *P* < 0.01 between the indicated groups.

Previous studies have reported that MSCs could repair ALI-induced impaired alveolar fluid clearance (AFC) by secreting KGF [20]. We next measured the concentration of KGF in BALF and culture supernatant of hBMSCs. Consistent with several studies, the secret of KGF by hBMSCs was detected in the culture supernatant. Moreover, 1 day after injection of hBMSCs, the levels of KGF in BALF were increased compared with control and SII groups (*P* < 0.05) (Figure 7(a)).

MSC could downregulate expressions of proinflammatory factors to protect the host from extraordinary inflammatory damage [18]. Our results found that systemic treatment with hMSCs could significantly decrease the levels of TNF-*α* in BALF (Figure 7(b)), which may contribute to downregulating inflammatory responses and tissue injury.

## 4. Discussion

In China, ALI caused by smoke inhalation is the most common cause of death among victims of fire tragedies. At least 85% of deaths from fire disasters occur because of excessive inhalation of smoke and toxic gases [28]. Characterized by acute onset, rapid progression, severe illness, and high mortality, severe respiratory disease resulting from smoke inhalation is commonly seen in clinical practice [29].

ALI is a severe pathological condition clinically characterized by respiratory distress, refractory hypoxemia, and noncardiogenic pulmonary edema. A number of factors can lead to the development of ALI; these include sepsis, pneumonia, trauma, aspiration of gastric contents, and exposure to large amounts of smoke from fires [30]. Smoke inhalation-induced ALI has unique pathophysiological features that differ from ALI caused by sepsis or pneumonia. Components found within smoke, including particulate materials, systemic toxins, and respiratory irritants, trigger the production of a cascade of inflammatory mediators within the airway mucosa and lung parenchyma, causing damage to mucosal lining and leading to peribronchial inflammation, which ultimately can result in pulmonary edema and ventilation/perfusion mismatch [1, 3–5]. During this process, intrapulmonary leukocyte aggregation following activation of the classic complement cascade releases even more chemokines and cytokines, leading to airway cast formation and widespread plugging. Moreover, the resultant induction of nitric oxide (NO) synthase in respiratory epithelial cells and alveolar macrophages leads to NO production, which increases bronchial blood flow, decreases hypoxic pulmonary vasoconstriction in poorly ventilated regions within the lung, and results in ventilation/perfusion mismatch. NO also forms peroxynitrite (ONOO^−^) by combining with superoxide (O_2_^−^) produced by neutrophils, which can lead to DNA damage and alveolar epithelial cell death [4, 5].

As with the other factors that cause ALI, efficient and specific therapies are needed for smoke inhalation-induced ALI. To develop these therapies, additional studies are needed because the pathological mechanisms underlying ALI remain poorly understood, and the current supportive methods used to treat the condition, including basic mechanical ventilation, fluid resuscitation, and oxygen administration, are not as effective as desired [1, 4, 6]. Different types of cell therapies are expected to have the ability to cure a wide variety of diseases, substantially improving the routine therapies currently used in the clinic [31]. Due to their low expression levels of immune antigens, MSCs are an attractive cell resource for the treatment of various complicated and refractory diseases [32]. Preclinical studies in small (mouse and rat) and large (sheep) animal models, as well as ex vivo studies using perfused human lungs, have demonstrated the potential efficacy and safety of MSC administration for the treatment of ALI/ARDS [17–20, 33]. In the current study, we demonstrated that hMSCs exert a protective effect on mice with smoke inhalation-induced ALI, suggesting that these cells may offer a therapeutic strategy for SII.

Despite recent interest in the use of adult stem cell therapy due to the multipotent nature of bone marrow-derived stem cells, findings regarding the engraftment process of systemically administered hBMSCs in lung injury models have varied. The majority of studies on experimental lung injuries have demonstrated an MSC engraftment rate of less than 1–5% [34–36]. In the current study, using BLI, we tracked the distribution dynamics of systemically administered hBMSCs in immunodeficient mice with SII. The results showed that a greater number of hBMSCs were found in the lung tissues of SII mice compared with control mice, a finding that is similar to previous studies [21] of mice exposed to trauma or bearing tumors. Our results further confirmed that hBMSCs innately traffic to sites of inflammation, which are concordant with previous studies of MSC localization patterns in trauma, cancer, and following exposure to radiation [37–39]. However, we found no hBMSC engraftment in the lung, which was demonstrated by the complete disappearance of BLI signal at 14 days after hBMSC administration. This result indicates that engraftment of the cells into the lung is not a major driving force behind the beneficial effects that were noted.

Growing evidence has indicated that the effects of MSCs with regard to lung tissue repair are not attributable to the differentiation capacity of these cells but rather to their activation of a protective mechanism and their stimulation of endogenous regeneration factors [18, 40]. MSCs produce soluble bioactive factors known to reduce alveolocapillary membrane permeability, inhibit apoptosis and fibrosis, decrease inflammation, and enhance tissue repair. Impaired AFC (alveolar fluid clearance) is common in patients with ALI/ARDS and leads to pulmonary edema. BMSCs also produce several epithelial growth factors, including vascular endothelial growth factor (VEGF), keratinocyte growth factor (KGF), and hepatocyte growth factor (HGF). KGF has been shown to improve alveolar fluid transport in part by upregulating aENaC gene expression [41] and Na-K-ATPase activity [42]. As such, KGF is important in the resolution of lung injury. In a set of ex vivo experiments, Matthay MA and colleges found that siRNA-mediated inhibition of KGF expression decreased the beneficial effects of MSCs in restoring AFC in injured, perfused human lungs by approximately 80% [19]. In the current study, we found that MSCs significantly attenuated pulmonary edema induced by smoke inhalation. The paracrine secretion of KGF leading to the restoration of AFC may be a possible mechanism for this finding.

The immunomodulatory effects of MSCs are well-established, and MSCs exert protection against inflammatory damage by downregulating the expression of proinflammatory factors, such as IL-1b, IL-8, interferon- (INF-) *γ*, and TNF-*α* [43]. MSCs also secret anti-inflammatory agents such as IL-4 and IL-10 to regulate the development of lung inflammation [44], reduce neutrophil infiltration into the lung, and reduce the quantity of proinflammatory cytokines in circulation, collectively maintaining a balance between inflammatory and anti-inflammatory responses [45]. Our results showed that hMSCs could significantly decrease the levels of TNF-*α* in BALF after 1 day after injection. Correspondingly, smoke inhalation resulted in inflammatory cell infiltration around the airway, which was significantly attenuated by the administration of hBMSCs. Thus, immunomodulation plays an important role in downregulating inflammatory responses and attenuating tissue injury in SII.

In summary, our study demonstrated that systemically administered hBMSCs mainly localized to the lungs of mice with SII. The hBMSCs attenuated the lung injury induced by smoke inhalation, and this may be due to the effect of anti-inflammatory and secreting KGF of hMSCs but not associated with the differentiation potential of the cells.
